# Philadelphia Medical Society

**Published:** 1841-11-06

**Authors:** 


### PHILADELPHIA MEDICAL SOCIETY.

*Saturday, Nov. 6th.*—The Medical Society held the first meeting of its annual session. The Society proceeded to organise itself, and appoint the several standing committees and chairmen. The Committee on Lectures of the preceding session reported the following arrangement of the order of addresses for the present winter.

Drs. R. M. Huston—On Vaccination as a preventive of Small Pox.
Warrington—On Diseases within the Female Pelvis producing symptoms analogous to those of displacement of the Uterus.
R. Coates—On Animal Magnetism.
Bryan—On Diseases of the Joints.
Parrish—On Fractures involving the Elbow and Wrist Joints.
West—On the evidences of Mineral Poisons taken into the Stomach.
Hallowell—On Cholera Infantum.
H. S. Patterson—On the use of cold water in disease.
Hays—On Scrofulous Ophthalmia.
Griscom—On Vaccination.
Pepper—On Pneumothorax.
Wiltbank—On Medical Ethics.
C. W. Morris—On the nature and treatment of Croup.

